# Physical Activity and Exercise Addiction During the Covid-19 Pandemic in Italy

**DOI:** 10.1007/s11469-022-00815-z

**Published:** 2022-04-20

**Authors:** Franca Ceci, Francesco Di Carlo, Julius Burkauskas, Anatolia Salone, Ilaria De Luca, Dorotea Cicconcelli, Valentina Giorgetti, Irene La Fratta, Antonino Todaro, Pierluigi Simonato, Giovanni Martinotti, Massimo di Giannantonio, Ornella Corazza

**Affiliations:** 1 Department of Neuroscience, Imaging, Clinical Sciences, University G. d’Annunzio of Chieti-Pescara, Chieti, Italy; 2 Laboratory of Behavioral Medicine, Neuroscience Institute, Lithuanian University of Health Sciences, Kaunas, Lithuania; 3 Department of Clinical and Pharmaceutical Sciences, University of Hertfordshire, Hatfield, UK; 4 Department of Medical and Oral Sciences and Biotechnologies, University G. d’Annunzio of Chieti-Pescara, Chieti, Italy; 5 IRCCS Fatebenefratelli, Brescia, Italy; 6 Department of Medico-Surgical Sciences and Biotechnologies, Sapienza University of Rome, Rome, Italy

**Keywords:** Exercise addiction, Appearance anxiety, Self-compassion, Performance and image enhancing drugs, Covid-19

## Abstract

Severe restrictive measures were implemented globally to limit the spread of the Covid-19 pandemic leading to significant lifestyle changes and impacting on both the physical and the mental health of citizens. Caught by the fear of getting sick, some individuals have adopted behaviors which favored the development of exercise addiction (EA). Our aim was to evaluate physical activity habits and the risk of EA in the general Italian population during phase 1 of the lockdown. The role of appearance anxiety (AA), self-compassion, and use of performance and image enhancing drugs (PIEDs) as predictors of EA development were investigated. A comparison between physically active subjects with the inactive ones was also included. Between April and May 2020, an online survey was conducted across Italy. Nine hundred thirty-six answers were collected. The rate of EA in the physically active sample (782 subjects) was 4.1%. The physically active group showed higher SCS scores and a greater use of PIEDs. Of the physically active participants, 84.2% reported variations in their fitness routine. Perceived benefit of exercising resulted significantly higher in those with EA. Subjects with EA reported stronger motivation in engaging in physical activity as for “physical wellness,” “psychological well-being,” and “sexual attractiveness and confidence in relationship.” A higher level of AA, a lower level of self-compassion, and a higher perceived benefit of exercising during lockdown were all significant predictors for the presence of EA. Our findings suggest that the fear of getting sick from Covid-19, combined with radical changes in the lifestyles induced by the lockdown and individual personological characteristics, can favor the development of EA and related phenomena in the general population.

On March 11, 2020 the World Health Organization (WHO) declared the beginning of the COVID-19 (COrona VIrus Disease − 19) pandemic caused by the new coronavirus SARS-CoV-2. Governments and health authorities of all the world took emergency measures to contrast the spread of the virus (Eubank et al., 2020) leading to significant changes in the lifestyles of the entire world population (di Renzo et al., 2020; Hu et al., 2020), while impacting on their social and economic lives (Dubey et al., 2020) and physical and mental health (Hossain et al., 2020). Some of these measures have also been implemented during other epidemics in the past (Brooks et al., 2020; Jeong et al., 2016), but the global occurrence of this pandemic could have more serious and different effects than those observed previously (Holmes et al., 2020; Horesh & Brown, 2020).

On the same day, with the implementation of the “IoRestoaCasa” (“StayatHome”) Decree of the President of the Council of Minister, phase 1 of the Italian lockdown also began and lasted until 3 May 2020 (Governo Italiano Presidenza del Consiglio dei Ministri, 2020). Italy was the first country in the EU to register cases of Covid-19 infection (Ziniti, 2020) and this measure regulated the immediate hard closure, throughout the national territory, of all activities (with the exception of hospitals, grocery stores, pharmacies, para-pharmacies, newsagents, and tobacconists) and introduced smart working and remote training for all the others. It was possible to leave one’s home, only after signing a self-certification document, exclusively for reasons of health, work, and for the purchase of necessary goods (Governo Italiano Presidenza del Consiglio dei Ministri, 2020). In the same month, the WHO launched the hashtag #HealthyAtHome with the aim of providing advice to better deal with the changes induced by the lockdown in lifestyle and habits and reduce the stress, depression, and anxiety associated with crisis period in progress (World Health Organization, 2020). Among the many tips, it was recommended to exercise regularly and stay active indoors, while gym, swimming pools, and fitness centers were closed, and public spaces were not accessible for physical exercise (Lim, 2021). We know that such drastic measures facilitated sedentary lifestyles and unhealthy eating habits (Ammar et al., 2021). Even among those who practiced good levels of physical activity before the pandemic, there was a significant reduction in this healthy habit and the development of a more sedentary lifestyle (Castañeda-Babarro et al., 2020).

Exercise has numerous health beneficial effects and is therefore usually considered a positive behavior (Garber et al., 2011; Peluso & Guerra de Andrade, 2005). Studies conducted during this pandemic even showed how a correct practice of it could even decrease hospitalization rates and mortality in patients with Covid-19 (Crisafulli & Pagliaro, 2020). More generally, it has also been shown that an increase in physical activity led to an improvement of quality of life (Sadeghipour et al., 2021). However, there are some controversies regarding this, as some individuals who habitually practiced physical activity before the pandemic have been negatively affected by the limitations imposed, developing sleep disturbances and less well-being (Martínez-de-Quel et al., 2021).

In addition to its positive side however, physical activity can also be associated with impaired mental health, being related to disturbances like “excessive exercise” and “overtraining syndrome” (Hausenblas & Downs, 2002; Peluso & Guerra de Andrade, 2005).

Back in 1979, Morgan observed that exaggerated physical exercise could lead to both physical injury and the failure to fulfill daily responsibilities such as those related to work and family, going to take the shape of a new type of addiction. It was associated with withdrawal symptoms, problems with daily functioning, exercise practice despite medical contraindications, and interference with social relationships and work (Morgan & O’Connor, 1988). Thus, the term “exercise addiction” (EA) began to be used (Griffiths, 1996; Szabo, 2009). Hausenblas and Downs (Downs et al., 2004; Hausenblas, 2002) characterized EA based on criteria, which are modifications of the DSM-IV TR (American Psychiatric Association., 2000) ones for substance dependence.

EA is not officially included in any of the international classifications of mental disorders but, based on its leading symptoms, it could be potentially classified in the category of behavioral addictions (Albrecht et al., 2007; Grant et al., 2010; Griffiths, 1996). Epidemiological distribution of EA is very heterogeneous. This could be due to two reasons: psychometric tools used in the assessment and the target population studied.

The incidence of EA is high in professional athletes, with rates of 25% in runners (Slay et al., 1998) and 30% in triathletes (Blaydon & Lindner, 2002). In members of fitness clubs, it is about 12%. Observational studies showed instead a rate ranging from 0.3 to 0.5% in the general population; among regular exercisers, it ranged from 1.9 to 3.2% (Griffiths, 2005; Mónok et al., 2012).

Contemporary society acts as an important reinforcer in EA, as health and education organizations along with the media support the beneficial effects of exercise and thinness. For this reason, this form of addiction is one of the most “hidden” behavioral addictions (Berczik et al., 2012). In our society, there is a very strong correlation between the idea of a “perfect” body and success in life (Mooney et al., 2017). This in some cases can lead to the development of a real appearance anxiety (AA). If particularly severe, it can led to the development of various appearance-related disorders, such as body dysmorphic disorder (BDD) (Al-Sarraf et al., 2018; Buhlmann et al., 2010) and muscle dysmorphia (MD) (Sandgren & Lavallee, 2018). BDD is classified under the DSM-5’s obsessive-compulsive and related disorders, and MD is a specifier of it (American Psychiatric Association, 2013). Formerly referred to as dysmorphophobia, BDD is a serious psychiatric condition characterized by a recurring and persistent concern about an imaginary or minor defect in physical appearance; this can regard a specific part of the body (the nose, hair, freckles, breast size) or the body as a whole (Buhlmann et al., 2009). Concerns are intrusive, unwanted, and usually difficult to control (Bewley, 2017; Silver & Farrants, 2016). These include compulsive looking in the mirror, which affects most people with BDD (Veale & Riley, 2001). BDD is underestimated and often undiagnosed (Bewley, 2017). When untreated, it affects most aspects of a person’s life and ultimately the global psychological well-being, as evidenced by its frequent association with severe depression, eating disorders (ED), addictive behaviors, suicidal ideation, and functional impairment (Altamura et al., 2001; Beucke et al., 2016; Leone et al., 2005; Murray et al., 2012; Soler et al., 2018; Veale & Bewley, 2015). Moreover, subjects affected by EA and BDD are particularly vulnerable to the use of performance and image enhancing drugs (PIEDs) (Corazza et al., 2019), increasingly sold on the web through misleading marketing strategies. PIEDs refer to a wide range of products, which are presented as having the potential to improve mental and physical functions. They include drugs for enhancing muscle structure and function, for weight loss, for modifying the aging process, beauty, and cosmetic appearance, for improving sex performance, for cognitive performance, among other functions. The market of PIEDs is poorly regulated (Corazza & Roman-Urrestarazu, 2017; Reuter & Pardo, 2017; U.S. Government Accountability Office, 2002), and these products are frequently contaminated with biologically active ingredients, so that they can pose significant health risks to those who use them (Graham et al., 2009; Mooney et al., 2017; Thevis et al., 2008; van de Ven et al., 2018).

On the contrary, one element that has been shown to be protective of the development of EA, BDD, and excessive use of PIEDs is “self-compassion” (Castilho & Gouveia, 2011). It is an emotional self-regulation strategy associated with self-acceptance and self-nourishment capabilities, which could be a protective factor for the development of previous disorders (Costa et al., 2016).

The radical changes that the Covid-19 pandemic brought to people’s daily lives created a fertile ground for the development of different forms of addiction (Czeisler et al., 2020; Martinotti et al., 2020). This is well known for substance abuse, while the implications these changes may have had on EA, body image disturbances, and use of PIEDs are less clear. Considering the reduced possibility of exercising during the pandemic, together with the fewer daily stressors, it could be hypothesized that during this period, people may have had more relaxed training rhythms and a lower AA, resulting in less use of PIEDs. In the past, however, studies among young adults documented that daily events were rated more stressful during social isolation (Cacioppo & Hawkley, 2003). It is therefore also possible that the practice of compulsive physical exercise (which can however be done at home or in open spaces facing the home) and the use of PIEDs became coping strategies to address the radical restrictions imposed by the lockdown. This could also be related to the higher amount of time spent on the Web during the lockdown, with numerous news and advertisements relating to body image, sports performance, fitness, and PIEDs.

With our study, we aimed to assess the risk of EA in physically active subjects within the general population during the strict phase 1 lockdown in Italy and investigate the motivations that pushed at risk individuals to train in an excessive way. We also evaluated AA, self-compassion, and use of PIEDs in our sample, testing for associations between those predictors and the risk of EA. Finally, we aimed to compare the physically active subjects with the physically inactive ones, to better characterize the role of physical activity during the pandemic.

## Methods

### Design

This is a cross-sectional study. An online survey was conducted across Italy from April to May 2020, a time frame precisely coinciding with the strict lockdown period due to the Covid-19 pandemic in that country.

### Sample

A total of 953 participants were recruited in the study. They were mainly women (66%), with a mean age of 31.54 ± 10.35 years. A total of 782 (82.1%) of the sample practiced physical activity. A significant part of our sample was composed by key workers, i.e., those workers who are essential for the country, as police, school facilities, and health care services (28.9%, *n* = 275). 20.5% (*n* = 195) of participants self-reported a mental disorder before the pandemic, mainly anxiety disorders.

### Instruments

The survey was composed of general socio-demographic information, questions about sport practice and about motivations in engaging in physical activity, a question about the use of PIEDs, and three psychometric scales: the Exercise Addiction Inventory (EAI), the Appearance Anxiety Inventory (AAI), and the Self-Compassion Scale (SCS).

Socio-demographic and general information included age, gender, job and education status, being a key worker, to have a mental disorder before the pandemic, and if the lockdown worsened this disorder.

Questions about physical activity were posed to better characterize the subjects with possible EA. These included types of sport practiced before the pandemic, variation of the fitness routine during the social distancing, assessed through a yes/no question, and perceived benefit of exercising during this period, measured on a 0–10 visual analogue scale (VAS). Different motivations in engaging in sport were assessed through a 1–5 VAS scale. Finally, the use of PIEDs was assessed.

The Exercise Addiction Inventory (EAI) (Kovacsik et al., 2019; Terry et al., 2004) was developed to assess the level of engagement in physical activity. It is a 6-item questionnaire that investigates six general components of addiction (salience, mood modification, tolerance, withdrawal symptoms, social conflict, and relapse). Each response is rated from 1 (strongly disagree) to 5 (strongly agree, with maximum score of 30). A score of 24 or higher is suggestive of probable EA. This cut-off was chosen as it represented the subjects scoring in the top 15% of the total scale score in the original study. The scale showed a Cronbach’s alpha of 0.84 and a test-retest reliability of 0.85 (Griffiths, 2005; Griffiths et al., 2015; Terry et al., 2004). The Italian version of the scale demonstrated acceptable internal consistency (alpha = 0.71) (Gori et al., 2021). The coefficient in the present sample was 0.751, suggesting an acceptable internal consistency.

The Appearance Anxiety Inventory (AAI) (Veale et al., 2014) assesses anxiety about body image and can be useful in the diagnosis of BDD. It is a 10-item questionnaire assessing frequency of avoidance and monitoring behaviors, as repeatedly checking in the mirror. In its original version, each item is rated from 0 (not at all) to 4 (all the time). In our version, items were from 1 (not at all) to 4 (all the time), with a total score of 40. Higher scores at the AAI indicates higher levels of appearance anxiety. The scale originally showed a Cronbach’s alpha = 0.91, and a test-retest reliability of 0.87. Cross-cultural studies including the Italian version of the scale showed a coefficient between 0.80 and 0.84 (Dores et al., 2021). The Cronbach’s alpha in the present sample was 0.878, suggesting a good internal consistency.

The Self-Compassion Scale (SCS-Short Form) (Neff, 2003) assesses self-compassion as a specific strategy to deal with emotional self-regulation. It is a 12-item questionnaire organized into six domains: self-kindness, self-judgment, common humanity, isolation, mindfulness, and over-identification. Each item is rated from 1 (almost never) to 5 (almost always). Total score is 60 points, with higher scores indicating a greater self-compassion. The original scale showed Cronbach’s alpha of 0.92. The Italian version of the scale showed a Cronbach’s alpha between 0.79 and 0.90 for the different domains (Veneziani et al., 2017). The Cronbach’s alpha coefficient in the present sample was 0.802, suggesting a good internal consistency.

### Procedure

The web-based survey platform Qualtrics (Qualtrics, Provo, UT, 2020 Available at https://www.qualtrics.com) was used to collect data. The survey was disseminated through social media platforms (Facebook, Twitter, WhatsApp, Instagram) and through the snowball sampling technique, consisting in inviting participants to share themselves the survey to their contacts. Participation to the survey was anonymous and free of compensation. After the recruitment, data were securely stored in an Excel database at the University of Hertfordshire, Hatfield, UK.

### Statistical Methods

The SPSS for Windows version 17.0 (SPSS Inc., Chicago, Illinois) was used for statistical analysis. Normality checking yielded adequate values. Data were expressed as mean and dispersion measures or as frequency and percentage, as appropriate. Student’s t-tests and Mann-Whitney U tests were calculated to compare continuous variables between the subsamples of EA and non-EA participants. Chi-square tests and Fisher exact tests were used for comparing categorical variables. Binary logistic regression was calculated to inspect how age, gender, perceived benefit of exercising during the lockdown, AAI, SCS, and use of PIEDs predict the risk of EA. Significance was set for *p* < 0.05.

### Ethical Considerations

The study was approved by the Human Sciences Ethics Committee at the University of Hertfordshire (HSK/SF/UH/00104), and by the Ethics Committees of each participating country. It complied with the Declaration of Helsinki and with the European General Data Protection Regulation.

## Results

### Psychometric Assessment

The whole sample (953 subjects) was made up of 82.1% of subjects practicing physical activity and 17.9% of subjects not practicing physical activity.

The rate of EA in those practicing physical activity was 4.1% (*n* = 32). Mean EAI score was 25.13 ± 1.16 in the subsample with EA and 16.56 ± 3.49 in the subsample without EA.

As to psychometric assessment, AAI scored significantly higher in subjects at risk of EA (*p* = 0.001), with a mean score of score of 19.03 ± 6.64; this mean score is close to 21, which may be indicative of a high risk of BDD. On the contrary, SCS scores were higher in the group without EA (*p* < 0.001). The use of PIEDs was reported significantly more in the subsample at risk of EA (*p* < 0.001). Detailed socio-demographical and psychometric data are reported in Table 1.

**Table 1:** Demographic characteristics and psychometric assessment

	Total *N* = 953	Total of physically active individuals *n *= 782	EAI ≥ 24 *n* = 32	EAI < 24 *n* = 750	Statistic	*p*	Effect size
Age	31.54 ± 10.35	31.87 ± 10.44	32.81 ± 11.03	31.83 ± 10.42	t = 0.521	0.602	*d* = 0.091
Sex *(n; %)*					χ^2^ = 0.560	0.454	
Men	324 (34.0%)	268 (34.3%)	9 (28.1%)	259 (34.5%)			*h*=−0.138
Women	629 (66.0%)	514 (65.7%)	23 (71.9%)	491 (65.5%)			*h* = 0.138
Physical activity, yes	782 (82.3)	782 (100%)	32 (100%)	750 (100%)			
Occupation					χ^2^ = 2.519	0.618	
Employed	477 (50.1%)	398 (50.9%)	14 (43.8%)	384 (51.2%)		0.409	*h*=−0.148
Student	240 (25.2%)	186 (23.8%)	7 (21.9%)	179 (23.9%)		0.796	*h*=−0.048
Unemployed	104 (10.9%)	84 (10.7%)	4 (12.5%)	80 (10.7%)		0.743	*h* = 0.056
Retired	126 (13.2%)	110 (14.1%)	7 (21.9%)	103 (13.7%)		0.195	*h* = 0.216
Freelance	6 (0.6%)	4 (0.5%)	0 (0%)	4 (0.5%)		0.689	*h*=−0.142
Education level (*n*; *%*)					χ^2^ = 0.226	0.956	
PhD	17 (1.8%)	14 (1.8%)	0 (0%)	14 (1.9%)		1.000	*h*=−0.277
Master’s degree	309 (32.4%)	260 (33.2%)	11 (34.4%)	249 (33.2%)		0.890	*h *= 0.025
Bachelor’s degree	208 (21.8%)	174 (22.3%)	8 (25.0%)	166 (22.1%)		0.703	*h* = 0.068
High school	419 (44.0%)	334 (42.7%)	13 (40.6%)	321 (42.8%)		0.806	*h*=−0.045
Key worker (*n*; *%*)	275 (28.9%)	227 (29.0%)	8 (25.0%)	219 (29.2%)	χ^2^ = 0.263	0.608	* h*=−0.095
Health care and related specialities	134 (48.7%)	107 (47.1%)	4 (50.0%)	103 (47.0%)	χ^2^ = 0.027	1.000	*h* = 0.060
Teachers and tutors	9 (3.3%)	8 (3.5%)	0 (0%)	8 (3.7%)	χ^2^ = 0.303	1.000	*h*=−0.387
Transportation	3 (1.1%)	2 (0.9%)	0 (0%)	2 (0.9%)	χ^2^ = 0.074	1.000	*h*=−0.190
Food Industry	13 (4.7%)	10 (4.4%)	0 (0%)	10 (4.6%)	χ^2^ = 0.382	1.000	*h*=−0.432
Public sector	1 (0.4%)	1 (0.4%)	0 (0%)	1 (0.5%)	χ^2^ = 0.037	1.000	*h*=−0.142
Government	14 (5.1%)	11 (4.8%)	1 (12.5%)	10 (4.6%)	χ^2^ = 1.054	0.332	*h* = 0.290
Postal and other services	10 (3.6%)	9 (4.0%)	1 (12.5%)	8 (3.7%)	χ^2^ = 1.587	0.280	*h* = 0.336
National or public security	7 (2.5%)	4 (1.8%)	0 (0%)	4 (1.8%)	χ^2^ = 0.149	1.000	*h*=−0.269
Pharmacy and related activity	9 (3.3%)	8 (3.5%)	0 (0%)	8 (3.7%)	χ^2^ = 0.303	1.000	*h*=−0.387
Mental disorder (before)	195 (20.5%)	147 (18.8%)	10 (31.3%)	137 (18.3%)	χ^2^ = 3.389	0.066	*h* = 0.307
Anxiety	91 (46.7%)	67 (45.6%)	3 (30.0%)	64 (46.7%)	χ^2^ = 1.050	0.347	* h*=−0.345
Depression	34 (17.4%)	24 (16.3%)	2 (20.0%)	22 (16.1%)	χ^2^ = 0.106	0.667	* h* = 0.102
Other mood disorders	15 (7.7%)	13 (8.8%)	0 (0%)	13 (9.5%)	χ^2^ = 1.041	0.601	*h*=−0.627
Eating disorders	25(12.8%)	21 (14.3%)	3 (30.0%)	18 (13.1%)	χ^2^ = 2.164	0.155	*h *= 0.419
Personality disorders	5 (2.6%)	4 (2.7%)	0 (0%)	4 (2.9%)	χ^2^ = 0.300	1.000	*h*=−0.342
Other(s)	25 (12.8%)	18 (12.2%)	2 (20.0%)	16 (11.7%)	χ^2^ = 0.601	0.353	*h* = 0.229
Addiction	94 (9.9%)	67 (8.6%)	2 (6.3%)	65 (8.7%)	χ^2^ = 0.229	1.000	*h*=−0.091
Physical distancing worsened mental disorder	74 (37.9%)	52 (35.4%)	5 (50.0%)	47 (34.3%)	χ^2^ = 1.004	0.325	*h *= 0.319
Psychometric assessment							
SCS	29.75 ± 5.22	30.03 ± 5.17	26.28 ± 5.26	30.19 ± 5.10	t=−4.240	< 0.001	*d* = 0.755
AAI	16.15 ± 5.36	15.96 ± 5.22	19.03 ± 6.64	15.83 ± 5.12	t = 2.699	0.001	*d *= 0.540
Use of PIEDs, yes	249 (26.1%)	234 (29.9%)	18 (56.3%)	216 (28.8%)	χ^2^ = 11.028	0.001	* h* = 0.564

Data are expressed as mean ± standard deviation and as *n *(%), as appropriate. Statistics: Student’s t-test, Chi-square test, Fisher exact test. *AAI*, Appearance Anxiety Inventory. *SCS*, Self-Compassion Scale. *PIEDS*, Performance and Image Enhancing Drugs

Finally, comparing the physically active subjects with the inactive ones, some differences could be highlighted: a higher percentage of physically inactive subjects reported a mental disorder before the pandemic (28.1% vs. 18.8%; *p* = 0.006) and addictive problems (15.8% vs. 8.6%; *p* = 0.004). The physically active group showed higher SCS scores (30.03 ± 5.17 vs. 28.46 ± 5.29; *p* < 0.001) and a greater use of PIEDs (29.9% vs. 8.8%; *p* < 0.001). Complete data are shown in Table 2.

**Table 2:** Physically active vs. physically inactive individuals

	Total *N* = 953	Physically active *n *= 782 (82.1%)	Physically inactive *n* = 171 (17.9%)	Statistic	*p*	Effect size
Age	31.54 ± 10.35	31.87 ± 10.44	30.05 ± 9.81	t = 2.090	0.037	*d* = 0.176
Sex *(n; %)*				χ^2^ = 0.145	0.703	
Men	324 (34.0%)	268 (34.3%)	56 (32.7%)			*h* = 0.034
Women	629 (66.0%)	514 (65.7%)	115 (67.3%)			*h*=−0.034
Physical activity, yes	782 (82.3)	782 (100%)	0 (0%)			
Occupation				χ^2^ = 7.723	0.091	
Employed	477 (50.1%)	398 (50.9%)	79 (46.2%)		0.269	*h* = 0.094
Student	240(25.2%)	186 (23.8%)	54 (31.6%)		0.035	*h*=−0.175
Unemployed	104 (10.9%)	84 (10.7%)	20 (11.7%)		0.706	*h*=−0.032
Retired	126 (13.2%)	110 (14.1%)	16 (9.4%)		0.103	*h* = 0.147
Freelance	6 (0.6%)	4 (0.5%)	2 (1.2%)		0.299	*h*=−0.078
Education level (*n*; *%*)				χ^2^ = 2.801	0.414	
PhD	17 (1.8%)	14 (1.8%)	3 (1.8%)		1.000	* h* = 0.000
Master’s degree	309 (32.4%)	260 (33.2%)	49 (28.7%)		0.258	*h* = 0.097
Bachelor’s degree	208 (21.8%)	174 (22.3%)	34 (19.9%)		0.495	*h* = 0.059
High school	419 (44.0%)	334 (42.7%)	85 (49.7%)		0.097	*h*=−0.141
Key worker (*n*; *%*)	275 (28.9%)	227 (29.0%)	48(28.1%)	χ^2^ = 0.063	0.802	*h *= 0.020
Health care and related specialities	134 (48.7%)	107 (47.1%)	27 (47.1%)	χ^2^ = 1.317	0.251	*h* = 0.000
Teachers and tutors	9 (3.3%)	8 (3.5%)	1 (2.1%)	χ^2^ = 0.260	1.000	*h *= 0.086
Transportation	3 (1.1%)	2 (0.9%)	1 (2.1%)	χ^2^ = 0.531	0.439	* h*=−0.101
Food Industry	13 (4.7%)	10 (4.4%)	3 (6.3%)	χ^2^ = 0.299	0.706	*h*=−0.085
Public sector	1 (0.4%)	1 (0.4%)	0 (0%)	χ^2^ = 0.212	1.000	*h *= 0.040
Government	14 (5.1%)	11 (4.8%)	3 (6.3%)	χ^2^ = 0.162	0.717	*h*=−0.066
Postal and other services	10 (3.6%)	9 (4.0%)	1 (2.1%)	χ^2^ = 0.400	1.000	*h* = 0.112
National or public security	7 (2.5%)	4 (1.8%)	3 (6.3%)	χ^2^ = 3.217	0.105	*h*=−0.238
Pharmacy and related activity	9 (3.3%)	8 (3.5%)	1 (2.1%)	χ^2^ = 0.260	1.000	*h *= 0.086
Mental disorder (before)	195 (20.5%)	147 (18.8%)	48 (28.1%)	χ^2^ = 7.412	0.006	*h*=−0.220
Anxiety	91 (46.7%)	67 (45.6%)	24 (50.0%)	χ^2^ = 0.284	0.594	*h*=−0.088
Depression	34 (17.4%)	24 (16.3%)	10 (20.8%)	χ^2^ = 0.511	0.475	*h*=−0.116
Other mood disorders	15 (7.7%)	13 (8.8%)	2 (4.2%)	χ^2^ = 1.115	0.367	*h* = 0.190
Eating disorders	25 (12.8%)	21 (14.3%)	4 (8.3%)	χ^2^ = 1.147	0.332	*h *= 0.191
Personality disorders	5 (2.6%)	4 (2.7%)	1 (2.1%)	χ^2^ = 0.059	1.000	*h* = 0.039
Other(s)	25 (12.8%)	18 (12.2%)	7 (14.6%)	χ^2^ = 0.177	0.674	*h*=−0.071
Addiction	94 (9.9%)	67 (8.6%)	27 (15.8%)	χ^2^ = 8.231	0.004	*h*=−0.222
Physical distancing worsened mental disorder	74 (37.9%)	52 (35.4%)	22 (45.8%)	χ^2^ = 1.681	0.195	*h*=−0.212
Psychometric assessment						
SCS	29.75 ± 5.22	30.03 ± 5.17	28.46 ± 5.29	t = 3.598	< 0.001	*d *= 0.305
AAI	16.15 ± 5.36	15.96 ± 5.22	16.82 ± 5.91	t=−1.763	0.079	*d*=−0.161
Use of PIEDs, yes	249 (26.1%)	234 (29.9%)	15 (8.8%)	χ^2^ = 32.524	< 0.001	*h *= 0.555

Data are expressed as mean ± standard deviation and as *n *(%), as appropriate. Statistics: Student’s t-test, Chi-square test, Fisher exact test. *AAI*, Appearance Anxiety Inventory. *SCS*, Self-Compassion Scale. *PIEDS*, Performance and Image Enhancing Drugs

### Physical Activity and Fitness Routine

Data about physical activity were analyzed in the sample reporting practicing sport (*n *= 782). The types of sport practiced did not differ between EA and non-EA subjects. The main type of sport reported in the sample was generic workout (43.2%). Regarding the impact of the pandemic on fitness routine, the social distancing appeared to have a strong effect, with 84.4% (*n *= 660) of the sample reporting variations in their routine. Perceived benefit of exercising resulted significantly higher in the participants at risk of EA (*p *< 0.001). Data about physical activity are reported in Table 3. EAI values for the participants from various sports were also investigated separately for men and women, as reported in Table 4.

**Table 3:** Psychometric assessment and physical activity habits

	Total *n *= 782	EAI ≥ 24 *n* = 32	EAI < 24 *N* = 750	Statistic	*p*	Effect size
Sports						
Generic workout	338 (43.2%)	15 (46.9%)	323 (43.1%)	χ^2^ = 0.181	0.670	*h* = 0.076
Running	128 (16.4%)	6 (18.8%)	122 (16.3%)	χ^2^ = 0.138	0.710	*h* = 0.066
Walking	93 (11.9%)	5 (15.6%)	88 (11.7%)	χ^2^ = 0.444	0.505	* h *= 0.114
Fighting sports	73 (9.3%)	2 (6.3%)	71 (9.5%)	χ^2^ = 0.375	0.760	*h*=−0.119
Martial Arts	35 (4.5%)	0 (0%)	35 (4.7%)	χ^2^ = 1.563	0.392	* h*=−0.437
Yoga	90 (11.5%)	2 (6.3%)	88 (11.7%)	χ^2^ = 0.906	0.569	* h*=−0.191
Swimming	70 (9.0%)	2 (6.3%)	68 (9.1%)	χ^2^ = 0.299	1.000	* h*=−0.105
Weight lifting	78 (10.0%)	5 (15.6%)	73 (9.7%)	χ^2^ = 1.186	0.276	*h *= 0.179
Cycling	54 (6.9%)	3 (9.4%)	51 (6.8%)	χ^2^ = 0.317	0.479	*h *= 0.096
Ball sports	59 (7.5%)	1 (3.1%)	58 (7.7%)	χ^2^ = 0.934	0.503	*h*=−0.208
Other	37 (4.7%)	3 (9.4%)	34 (4.5%)	χ^2^ = 1.596	0.189	*h *= 0.196
Dance	41 (5.2%)	4 (12.5%)	37 (4.9%)	χ^2^ = 3.537	0.080	*h* = 0.276
Mountaineering	31 (4.0%)	1 (3.1%)	30 (4.0%)	χ^2^ = 0.062	1.000	*h*=−0.049
Cross fit	24 (3.1%)	3 (9.4%)	21 (2.8%)	χ^2^ = 4.460	0.070	*h* = 0.287
Tennis	7 (0.9%)	0 (0%)	7 (0.9%)	χ^2^ = 0.301	1.000	* h*=−0.190
Triathlon	21 (2.7%)	2 (6.3%)	19 (2.5%)	χ^2^ = 1.622	0.211	* h *= 0.190
Variation of the fitness routine during the Social Distancing	660 (84.4%)	28(87.5%)	632 (84.3%)	χ^2^ = 0.244	0.805	*h* = 0.092
Perceived benefit of exercising during the Social Distancing	6.34 ± 3.02	8.68 ± 2.04	6.55 ± 2.75	t = 5.599	< 0.001	*d* = 0.880
min per week spent for a medium intensity training before COVID-19?	200 (140–320)	300 (185–420)	200 (120–300)	U = 8164.0	0.004	*d *= 0.221
How many months did you train in this intensity?	10 (4–24)	15 (8–24)	10 (4–24)	U = 8184.0	0.020	*d* = 0.219
min per week spent for a medium intensity training during COVID-19	180 (60–300)	245 (77.5–360)	180 (60–300)	U = 9641.5	0.070	*d* = 0.135

Data are expressed as mean ± standard deviation, median (25–75 perc.) and as *n* (%), as appropriate. Statistics: Student’s t-test, Mann-Whitney, Chi-square test, Fisher exact test

**Table 4:** EAI values for the participants from various sports separately for men and women

Sport Type	Men		Women		Statistic	*p*	*d*
	*N*	mean ± SD	*N*	mean ± SD			
Generic workout	101	16.08 ± 3.71	237	17.14 ± 3.68	t=−2.427	0.016	0.288
Running	54	16.76 ± 3.39	74	18.49 ± 3.48	t=−2.802	0.006	0.503
Walking	17	14.65 ± 3.72	76	15.49 ± 4.48	t=−0.719	0.474	0.193
Fighting sports	36	16.94 ± 3.53	37	17.41 ± 3.79	t=−0.538	0.593	0.126
Martial Arts	17	16.41 ± 3.48	18	17.17 ± 3.82	t=−0.609	0.546	0.206
Yoga	1	15.00	89	17.55 ± 3.26	U = 21.00	0.489	0.192
Swimming	22	16.27 ± 3.15	48	17.88 ± 3.97	t=−1.665	0.100	0.429
Weight lifting	36	18.31 ± 3.10	42	18.52 ± 4.01	t=−0.266	0.791	0.060
Cycling	26	16.65 ± 3.29	28	18.96 ± 4.38	t=−2.178	0.034	0.593
Ball sports	42	16.48 ± 3.23	17	18.24 ± 3.21	t=−1.896	0.063	0.546
Other	15	16.67 ± 5.86	22	17.82 ± 4.64	t=−0.666	0.510	0.223
Dance	1	20.00	40	18.15 ± 4.18	U = 13.0	0.683	0.186
Mountaineering	15	17.40 ± 3.07	16	18.44 ± 3.31	t=−0.904	0.373	0.325
Cross fit	5	20.80 ± 2.59	19	18.89 ± 4.25	U = 36.0	0.446	0.338
Tennis	4	16.25 ± 4.35	3	15.33 ± 2.08	U = 5.50	0.857	0.134
Triathlon	7	16.43 ± 2.57	14	20.43 ± 3.69	t=−2.556	0.019	1.185

Data are expressed as mean ± standard deviation. Statistics: Student’s t-test, Mann-Whitney

Different types of motivation in engaging in physical activity were investigated, to assess differences between the groups. Subjects at risk of EA reported stronger motivation as for “physical wellness” (*p *< 0.001), “psychological well-being” (*p *< 0.001) and “sexual attractiveness and confidence in relationship” (*p *= 0.001). See Table 5 for details.

**Table 5:** Motivation in engaging in physical activity

	EAI ≥ 24		EAI < 24		U	*p*	*d*
	*n* = 32		*n* = 750				
	Mean	Median (25–75 perc.)	Mean	Median (25–75 perc.)			
Physical appearance	3.25	3.0 (2.0–4.0)	3.10	3.0 (2.0–4.0)	11220.0	0.516	0.045
Physical wellness	3.56	4.0 (3.0–4.0)	3.12	3.0 (3.0–3.0)	7916.5	< 0.001	0.235
Psychological well-being	3.63	4.0 (3.0–4.0)	3.04	3.0 (3.0–3.0)	6816.0	< 0.001	0.300
General motivation (because I like to sport)	3.19	3.0 (3.0–4.0)	2.98	3.0 (2.0–3.0)	10339.5	0.162	0.095
Sexual attractiveness and confidence in relationship	3.59	4.0 (3.0–4.0)	3.05	3.0 (2.0–3.25)	7947.5	0.001	0.233
I feel that I am a part of sporting community	2.97	3.0 (2.0–3.75)	3.12	3.0 (2.0–5.0)	11685.5	0.795	0.018
I feel better than those who do not do sports	2.97	2.50 (2.0–5.0)	2.94	3.0 (1.0–5.0)	11667.0	0.778	0.019

Data are expressed as mean and median (25–75 perc). Statistics: Mann-Whitney U test

### Predictors of Exercise Addiction

Finally, a logistic regression model was built to assess predictor for the risk of EA in our sample recruited during the pandemic. Dependent variable was classified as 0 - EAI < 24 and 1 - EAI ≥ 24. Together with age and sex, the variables that resulted to be associated with EA in the previous analyses were inserted in the model. These were the AAI score, the SCS score, the use of PIEDs, classified as 0 – “no” and 1 – “yes,” and the level of perceived benefit of exercising during the social distancing. A higher level of AA, a lower level of self-compassion, and a higher perceived benefit of exercising during lockdown were all significant predictors for the risk of EA. Logistic regression model is detailed in Table 6.

**Table 6:** Logistic regression model to identify the risk for EA (EAI ≥ 24)

EAI ≥ 24 0 – no 1 – yes *n* = 782		B	Standard error	Odds ratio (OR)	Confidence interval (CI)		p
					Min	Max	
	Age	0.023	0.020	1.023	0.984	1.065	0.252
	Sex	−0.311	0.473	0.733	0.290	1.854	0.512
	AAI	0.082	0.036	1.085	1.012	1.164	0.022
	SCS total	−0.120	0.041	0.887	0.818	0.961	0.003
	PIEDs	0.777	0.408	2.175	0.977	4.843	0.057
	Perceived benefit of exercising	0.510	0.131	1.664	1.287	2.152	**< **0.001
	Constant	−5.662	2.159	0.003			0.009

*AAI*, Appearance Anxiety Inventory. *SCS*, Self-Compassion Scale. *PIEDS*, Performance and Image Enhancing Drugs

## Discussion

At present, there are still few data in the literature about the role played by the of the Covid-19 restrictive measures in facilitating risky coping behaviors such as EA or in influencing its etiopathogenetic and pathological aspects. However, interest in this topic is growing recently (Juwono & Szabo, 2020), because of the enormous changes that lockdowns caused in people’s life.

Our study finds a risk of EA during the Italian lockdown phase 1 of 4.1%, a value higher than those detected in previous studies performed on the general population (Griffiths et al., 2015; Mónok et al., 2012). In normal times, the risk of developing EA in these subjects is low, unlike for those who belong to fitness-related environments or are professional athletes. This is probably due to the fact that they are caught up in the numerous and hectic activities of daily life. The result we obtained instead may support our initial hypothesis that there are factors in this historical moment that can favor the development or exacerbation of this disorder that is usually characterized by a progressive development. The “four phases” model is the most widely recognized theory for explaining the development of EA: the first phase is recreational exercise, from there develops the risky exercise, then the problematic exercise, and finally the EA (Freimuth et al., 2011). This could be the progressive path to EA that occurs in both those who train for health reasons and those who use physical activity as a coping strategy. However, no assumption about the validity of this model in our sample can be drawn.

In our sample, no predisposing demographic factors for the development of EA were revealed, while a significant correlation was found between EA and having higher AAI and lower SCS scores. The subjects affected by EA would therefore have a greater AA and consequently a greater risk of developing related disorders such as BDD.

There is growing evidence that regular exercise improves immunity in individuals of all ages, and a physically active lifestyle can limit the aging of the immune system, reducing the risk of contracting both transmissible and not transmissible diseases (Campbell & Turner, 2018). Therefore, some individuals could push themselves to keep fit by exercising (Chamorro-Viña et al., 2014). This could be particularly the case in trying to prevent transmission of Covid-19, so that some people might adopt this new lifestyle by overdoing exercise and developing an unhealthy obsession with fitness. The blockade due to Covid-19 also offered a greater chance of over-exercising due to more flexible working hours from home, more free time available, reduced possibility to travel, and encouragement from public health organizations to get regular exercise. The same block also involved considerable stress, so physical activity could be a valid coping strategy, with the possible risk of triggering an addictive condition (Lim, 2021). In addition to conscious motivations, the unconscious motivation aimed at the perception of oneself as stronger and healthier must also be considered to reduce the subjective perception of risk of Covid-19 infection (Legrand, 2006).

Furthermore, some phenomena emerged to promote health and physical fitness can sometimes have opposite effects. One example is that of “fitspiration”, a new trend related to social media that push individuals to exercise to improve their health and appearance. Through the Internet, problems such as eating disorders, psychological distress, and addictive exercise behaviors can then be triggered or aggravated (Di Carlo et al., 2021; Raggatt et al., 2018). This can be of great significance at this time when social media is being used more than usual (Gottlieb & Dyer, 2020).

In this same period video chats are the main means of communication and work. A sufficient level of satisfaction with one’s appearance may represent a factor of considerable importance in this period (Pfund et al., 2020). In predisposed people, this could lead to the development of significant AA.

Another relevant finding that emerged from our survey is the higher consumption of PIEDs among those at risk for EA, although this is only a trend in the regression analysis. This is in line with the results provided by previous studies (Corazza et al., 2019). Moreover, the increased time spent online, searching for medical information could make these subjects vulnerable to purchasing PIEDs, advertised on the web as capable of drastically improving physical appearance faster and “safer” than traditional methods (Kamber & Mullis, 2010; Molinero, 2009; Müller, 2010).

We found no differences in terms of sport practiced between the group of subjects at risk of EA and that of non-at-risk subjects, therefore it does not seem that there is a sport (practiced at non-professional levels) that predisposes more than another to this disorder. The same result was found relatively to the variation of the fitness routine: the period of social distancing due to the pandemic from Covid-19 has led to important changes for everyone (de la Vega et al., 2020). Instead, what differentiates the two groups is the perceived benefit of performing physical activity, which is significantly higher in subjects at risk of EA, and the motivation that leads people to train. “Physical wellness,” “psychological well-being,” and “sexual attractiveness and confidence in relationship” are the reasons reported significantly more by the subjects at risk of EA. The different types of perceived benefits appear to be more harmonized in subjects with no risk of EA while they are more selective and related to the subjective perception of physical and mental well-being, rather than to comparison with other people, in subjects at risk of EA. This data could be in line with the greater predisposition to develop EA in subjects with greater perception of personal inadequacy and fragility and consequent compensatory use of physical exercise. The subjective self-perception and more precisely the bodily self-perception could represent a basic aspect predisposing the pathological evolution of the motivation to physical exercise (Legrand, 2006).

The differences found between the physically active and inactive groups suggest a protective role of physical activity against some mental disorders (Schuch et al., 2017; Stubbs et al., 2018), as well as a possible role in the development of good emotional self-regulation, as showed by higher SCS scores in those physically active. This latter positive relationship has been underlined by other studies (Sirois et al., 2015). The greater use of PIEDs in subjects practicing physical activity could instead underline a greater attention of those to physical shape and health, also sought with auxiliary means.

The results provided by the logistic regression emphasizes the role of AA as a risk factor for the development of EA and its strong association with BDD, whereas a good degree of self-compassion is an important protective factor. The trend between the use of PIEDs and being at risk of EA may also be relevant.

This study has however several limitations. The snowball recruitment process and the online nature of data limit the interpretation of the findings, as well as the presence of a non-stratified sample based on voluntary participation and the self-reported measures. The majority of the sample was made by women, possibly affecting the generalizability of the findings. Due to the self-reported nature of the data, some variables may lack of accuracy (e.g., presence of a previous mental disorder). Moreover, detailed information regarding the individual’s history of exercise and consumption of PIEDs is lacking.

## Conclusions

The prevalence of EA in a large sample of subjects recruited during the first strict lockdown period in Italy appeared higher than that detected in previous studies performed on the general population. A higher level of AA, a lower level of self-compassion, and a higher perceived benefit of exercising during lockdown were all significant predictors for the risk of EA. Therefore, despite some limitations, this study seems to suggest that EA, associated with phenomena such as BDD and the use of PIEDs, could have found fertile ground in a period of significant changes in the lifestyle habits. The findings highlighted in this study should lead to deepen these topics with further research, to make it possible to safeguard the physical and mental health in critical periods. It would be essential to guarantee targeted prevention strategies towards populations at risk, also making use of remote interventions appropriate to the pandemic period (Di Carlo et al., 2020) and to train clinicians and professionals operating in the sports scenario, to identify any potential subjects requiring referral to specialist care.

Longitudinal studies are needed to assess if the present findings will be preserved over time after the end of the pandemic phase.
